# Depositional patterns constrained by slope topography changes on seamounts

**DOI:** 10.1038/s41598-020-77573-2

**Published:** 2020-11-25

**Authors:** Dewen Du, Shijuan Yan, Gang Yang, Fengdeng Shi, Zhiwei Zhu, Qinglei Song, Fengli Yang, Yingchun Cui, Xuefa Shi

**Affiliations:** 1 Key Laboratory of Marine Geology and Metallogeny, First Institute of Oceanography, Ministry of Natural Resources, Qingdao, China; 2 Evaluation and Detection Technology Laboratory of Marine Mineral Resources, Qingdao National Laboratory for Marine Science and Technology, Qingdao, China; 3 Present Address: First Institute of Oceanography, Ministry of Natural Resources, No.6 Xianxialing Road, Laoshan District, Qingdao, 266063 China

**Keywords:** Ocean sciences, Planetary science, Solid Earth sciences

## Abstract

Slope topography is known to control the spatial distribution of deposits on intraplate seamounts; however, relatively little is known about how slope topography changes constrain those depositional patterns. In this study, we analyse data on four lithotypes found on seamount slopes, including colloidal chemical deposits comprising mainly cobalt-rich crusts, and examine the relationships between the spatial distribution of these lithotypes and current slope topography. We use these relationships to discuss depositional patterns constrained by slope topography changes. Some depositional units in drill core samples are interpreted to have resulted from past topographic changes that created the current slope topography. Two or more types of deposits that accumulated at the same location implies that the slope topography changed over time and that the depositional patterns on seamount slopes are constrained by changes in slope topography.

## Introduction

Seamounts are first-order deep-sea morphological elements^1^ and have important oceanographic research value. Cobalt-rich crust deposits that may contain several strategic metals and thus be considered mineral resources are widely distributed on seamount slopes^2–5^. Therefore, many scientists have surveyed and explored seamounts since the 1980s^6–9^, acquiring a large amount of data and knowledge on seamounts. However, some questions, such as how topographical changes to seamounts constrain depositional patterns on their slopes and how to interpret depositional sequences in sections of shallow drill samples taken from seamount slopes, remain unanswered.

**Figure 1:**
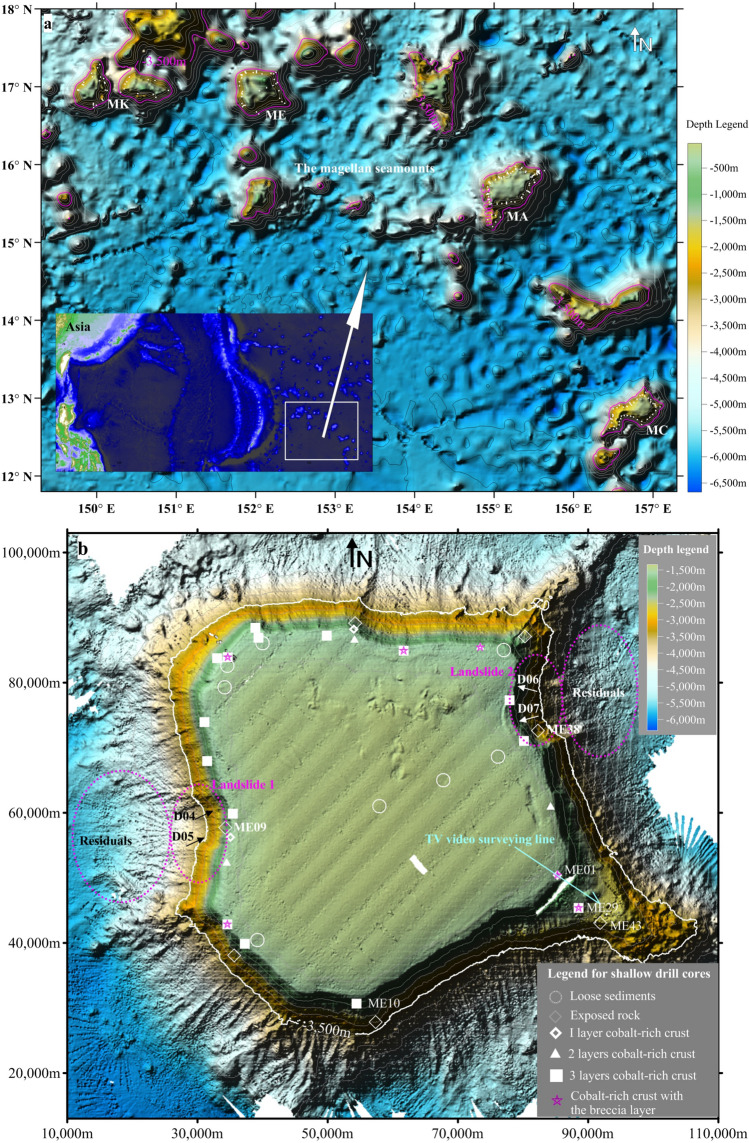
The Magellan Seamounts and locations of geological survey sites on them. (**a**) The map images are created using the SRTM30_PLUS^13^. Surveys acquired 205 shallow boreholes, including 82 from seamount MA, 51 from seamount MC, 37 from seamount ME and 35 from seamount MK, together with 20 TV grabs and 161 dredging samples on slopes between 1200 and 3500 m deep. Shallow drill sites are indicated by white dots shown in (**a**). Bathymetric data on the four seamounts covering an area of approximately 40,000 km^2^ measured using the EM122 Multibeam Survey System (Kongsberg, Inc.) by several survey cruises of the COMRA. Topography image of the seamount ME created using the bathymetric data is shown in (**b**). The TV video surveying line covering 18 km and two sizeable landslides on the flank of seamount ME are shown in (**b**), will be involved in the following.

The Magellan Seamounts in the northwest Pacific Ocean (Fig. 1) are typical intraplate seamounts that were active during the Late Cretaceous. Afterward, volcanic activity gradually ceased, and erosion reduced the seamounts to underwater structures that were subject to deposition and denudation^10^. The Magellan Seamounts are in a relatively stable tectonic position, which allows for comparatively undisturbed deposition and allows them to be general study objects for depositional patterns on their slopes. They are large cone-shaped extinct volcanoes, rising several kilometres above the abyssal seafloor, and most of the seamounts are between several tens to 100 km in diameter, as shown in Fig. 1. Their flat tops comprise a plateau-shaped summit covered by pelagic sediments^11^. Flanking slopes surround the flat tops with gradients ranging 0°–45°, with an average of approximately 15° and some slopes as steep as 60°. These slopes are widely covered by several lithotypes, mainly comprising cobalt-rich crusts and pelagic sediments^12^.

Cobalt-rich crusts are black ferromanganese deposits that are attached to substrate rocks^6,9,14^ such as basement outcrops or solid consolidated bedrock on summit boundaries and flank slopes. The distribution of these deposits is controlled by the carbonate compensation depth and minimum oxygen zone in the submarine environment^6^, i.e. depths of 800–3500 m, which lie within the slope region in this study. Pelagic sediments are relatively light in colour and comprise foraminiferal sand, silt and clay. Transitional zone sediments^15^ are a further important lithotype and are discussed in the next section. These unique lithotypes and their patterns differ from those of the deep-sea basin and continental margin environments^16,17^, and therefore warrant their own depositional models.

Many submarine environmental factors might constrain such depositional patterns^18–25^; however, geologic surveys have demonstrated that topography is one essential factor constraining their spatial distribution on seamount slopes^12,15,26–30^. Extinct seamounts are in relatively stable tectonic positions; however, they can also undergo topographic changes such as slope gradient adjustment, large area collapse or landslides on seamount flanks^11,31–33^. Although sediments disturbed by seamount topography changes have been observed, relatively little is known about how changes in slope topography constrain depositional patterns over the geological history of the seamount.

Over the past 10 years, the China Ocean Mineral Resources Association (COMRA) has conducted several survey cruises to study the Magellan Seamounts (Fig. 1) and has accumulated a large amount of data that are the focus of this study (Fig. 1; “Methods” section).

### Four lithotypes

**Figure 2:**
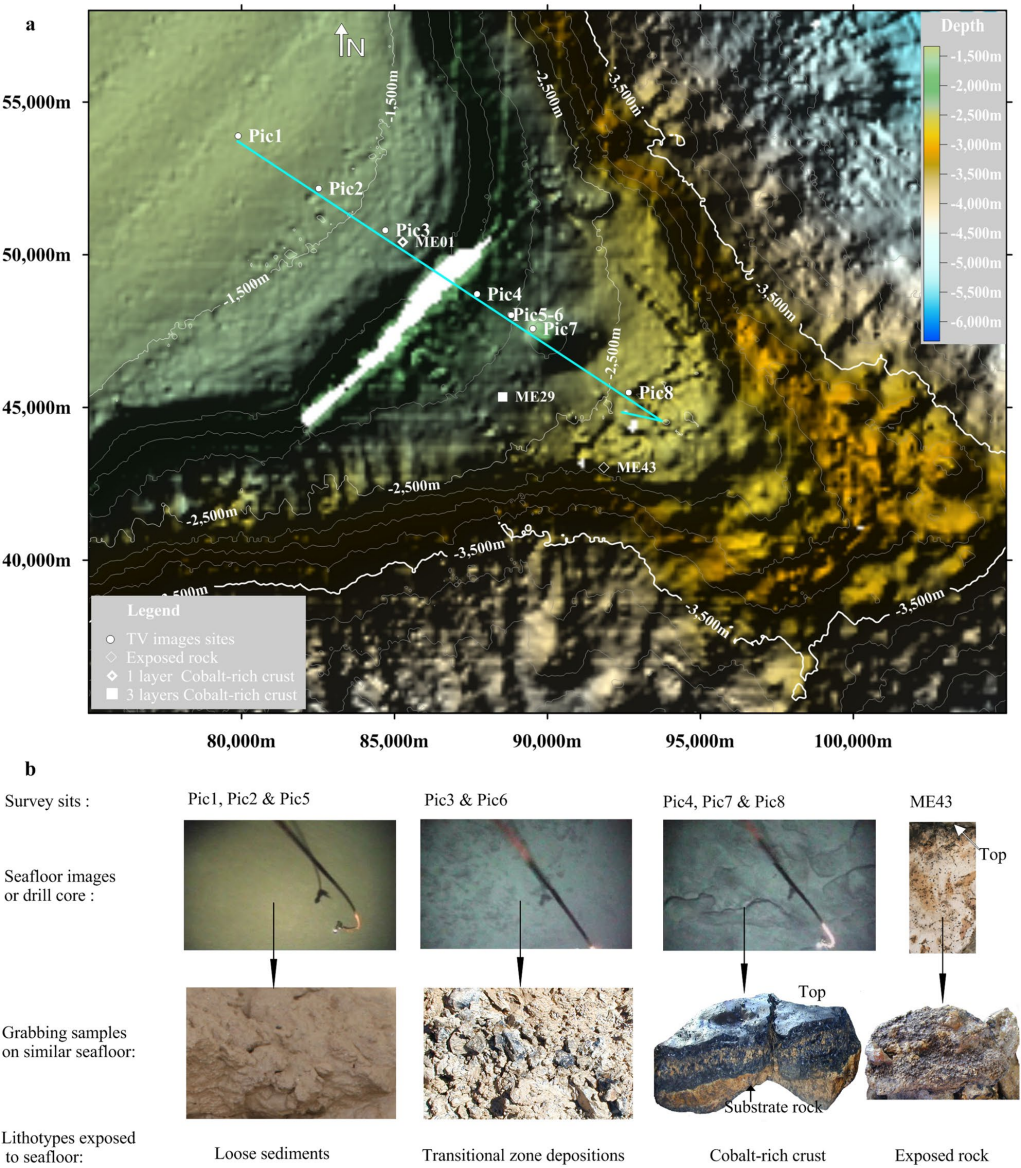
Four lithotypes on the slope of seamount ME. (**a**) Topography image of the southeast corner of the seamount ME and the 18 km-long TV video surveying line on it are also shown in Fig. 1b. (**b**) Representative images of the thousands of seafloor photos taken documenting the three lithotypes, i.e. loose sediments, transitional zone depositions and cobalt-rich crust. The video window is approximately 3 m × 5 m. The fourth lithotype in this study was exposed rock acquired by shallow drill at sampling site ME43.

Four distinct lithotypes exposed on the seafloor have been identified in this study. (1) *Exposed rock* such as volcanic breccias, basalt and consolidated sediments^34^ are exposed on seafloor and are not covered by any deposits. (2) *Loose sediments* are soft and unconsolidated and mainly comprise clay, silt and foraminiferal sands. These are interpreted as pelagic sediments, and are easily mobilised downslope by gravity processes^32,35,36^. (3) *Cobalt-rich crusts* comprise chemical colloidal deposits enriched in cobalt, phosphorite or calcium^37,38^. These occur as crusts attached to substrate rock surfaces that are relatively stable. (4) *Transitional sediments* accumulate on the transition zone separating slopes of different gradients. They are an unconsolidated mixture of rock debris, breccia, gravel, sand, clay, cobalt-rich crusts and ferromanganese nodules, as previously described by Yamazaki and Sharma^15^. Compared with loose sediments, transitional zone deposits experience a lower degree of mobilisation on seamount slopes. Similar observations have been presented by Yeo et al.^25^. All the lithotypes in this study are shown in Fig. 2. Except for exposed rock, each lithotype is a distinctive depositional facies.

Although the loose sediments on the flat tops of seamounts are similar to pelagic sediments everywhere, transitional zone sediments and cobalt-rich crusts on seamount slopes have distinct depositional patterns when compared with deep-sea basin and continental margin sediments. Additionally, the spatial distribution of the loose sediments, the transitional zone sediments and cobalt-rich crusts on seamount slopes are mainly constrained by seamount topography (i.e. slope gradient is a key factor).

## Results and discussion

### Slope gradients of lithotypes

**Figure 3:**
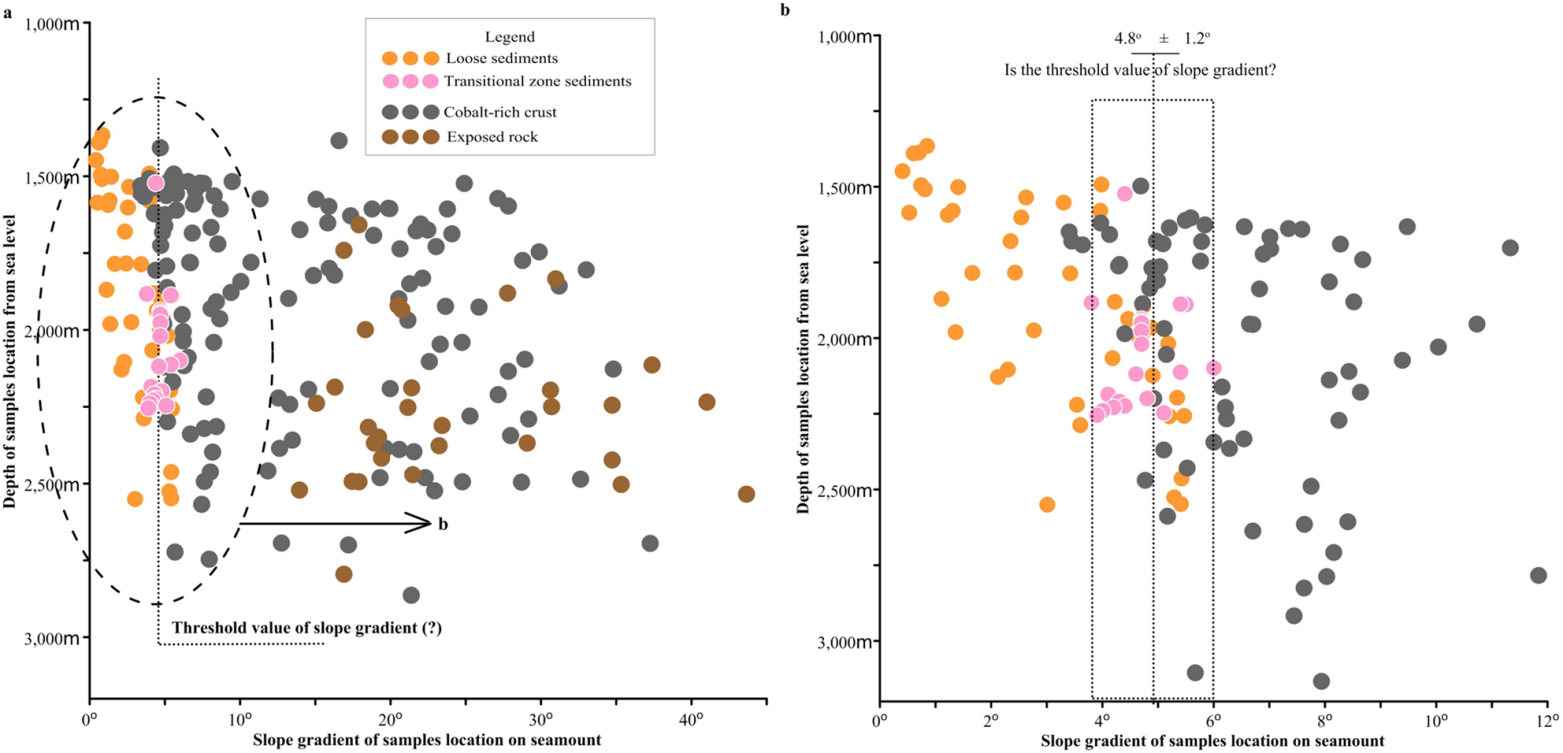
Relationships between slope gradients and types of lithotypes on the seamounts. (**a**) The sample sites are distributed mainly in 3300 m to 1500 m below sea level; the dashed vertical line represents the potential gradient threshold value of 4.8°. (**b**) 4.8° ± 1.2° is the potential threshold value range to control the distribution of loose sediments, transitional zone sediments, and consolidated units (cobalt-rich crust and exposed rock) respectively. Exposed rock samples, their number is 31, are on the slopes, with range of 15.1°–43.7°, and mean of 24.4° gradients; Cobalt-rich crust samples, their number is 132, are on the slopes, with range of 3.4°–37.3°, and mean of 13.9° gradients; Transitional zone depositions, their number is 20, are on the slopes, with range of 3.6°–6.0°, and mean of 4.0° gradients; Loose sediments, their number is 42, are on the slopes, with range of 0.4°–5.5°, and mean of 3.1° gradients.

The datasets involved in this study were obtained by geological sampling (e.g. shallow drilling and TV grabs) from 225 geological survey sites (Fig. 1a). Each dataset of the four lithotypes—loose sediments, transitional zone sediments, cobalt-rich crusts and exposed rock, includes information on the depth and slope gradient of the survey sites (Fig. 3). As shown in Fig. 3, loose sediments are generally confined to gentle slopes, whereas exposed rock and cobalt-rich crusts are preferentially distributed on steeper slopes and transitional zone sediments tend to appear on slopes with gradients between those characteristic of loose sediments and cobalt-rich crusts.

Similar observations can be found in the literature, and previous studies have identified slope topography’s role in controlling the spatial distribution of depositional patterns on seamount slopes. For instance, cobalt-rich crusts commonly form ring-shaped deposits on upper seamount slopes and along the periphery of the summit^12^. This contrasts with the flat tops and sharp escarpments of underwater seamounts, where the slope is less favourable for accumulating such deposits^26^. Typical seamount slopes can be divided into three zones: one dominated by nodules which are referred to one of the components of the transitional zone sediments here, with a gradient of up to 4°, one dominated by sediments with gradients of up to a 3°, and one with cobalt-rich crusts with gradients of up to 15°^15^. Shallow profilers and TV video have been used to delineate the upper boundaries for cobalt-rich crust deposition^27,39^. This is in addition to studies using shallow drilling and TV video to identify how micro-topography controls cobalt-rich crust enrichment^28^. Slopes greater than 20° are not suitable for growing cobalt-rich crusts^29^. However, slopes with cobalt-rich crusts all have gradients greater than 4° and slopes with loose sediment are all less than 4°^30^**.**

Deviations exist between different observations that could be caused by measurement error and cumulative influence of bottom flow, as elucidated by Stow et al.^23^; however, these observations consistently imply that current slope gradient controls the spatial distribution of lithotypes on slopes. Additionally, exposed rock carries other geological implications that will be explored in the next section.

### Intense slope topography changes and the depositional record

Huge slope collapses or landslides on the flank of seamount ME described by Smoot and King^40^ are typical topographic changes observed on seamounts around the world, and these events can be identified by several features^41^. The survey data shown in Table 1 provide a basis for evaluating landslide influence on slope depositions. Two drag samples (D04 and D05) and a shallow drilling sample (ME9) were taken from the body of landslide 1, as shown in Fig. 1b. Sample D04 comprises volcanic breccia with a manganese film, sample D05 consists of volcanic breccia and ME9 contains basalt without chemical encrustation. Two other drag samples (D06 and D07) and a shallow drilling sample (ME38) were taken from landslide 2, as shown in Fig. 1b. Sample D06 comprises volcanic breccia, sample D07 consists of limestone and ME38 contains volcanic breccia without chemical encrustation. The data reveals that besides residual slope depositions, the lithotypes underlying the landslides are primarily exposed rocks.

**Table 1:** Survey samples on or next to the landslides shown in Fig. 4 and their characteristics.

	Location	Depth (m)	Slope gradient (°)	Substrate rock	Crust thickness (mm)	Lithotypes	Landslide
Landslide 1 (Fig. 1b)	D05	2100	28.3	Volcanic breccia	0	Exposed rock	On
	D04	3074	22.6	Volcanic breccia	< 1	Exposed rock	On
	ME09	2351	36.2	Basalt	0	Exposed rock	On
	ME40	1563	5.6	Limestone	100	Cobalt-rich crusts	Peripheral to
	ME08	1659	3.1	Limestone	60	Cobalt-rich crusts	Peripheral to
Landslide 2 (Fig. 1b)	ME38	3040	16.9	Volcanic breccia	0	Exposed rock	on
	D06	1967	27.6	Volcanic breccia	0	Exposed rock	on
	D07	2100	21.1	Limestone	0	Exposed rock	on
	ME03	1623	4.3	Limestone	80	Cobalt-rich crusts	Peripheral
	ME37	1564	8.3	Limestone	90	Cobalt-rich crusts	Peripheral

Intense topographical changes on the slope, such as landslides, slope collapse and volcanic activity (which ceased in the Late Cretaceous in this case) on intraplate seamounts^10,42^, play a role in clearing deposits from seamount slopes and burying old deposits on slopes^25^. After past deposits have been removed, the seamount flank would consist exposed rock and would eventually experience build-up of new deposits.

Obviously, slope topography changes induced by either sudden collapse or gentle adjustments in slope gradient can influence deposition on the slope. For instance, landslides remove material from the slope in their proximal part and cover or disturb deposits in their distal part. When slopes undergo gentle adjustments in gradient, they become steeper, promoting growth of cobalt-rich crusts, or become gentler, inhibiting such growth. Therefore, this observation can be used as a basis to explore interactions between changes in seamount topography and deposition on seamount slopes in the geologic record.

### A stratigraphic record of slope topography changes

**Figure 4:**
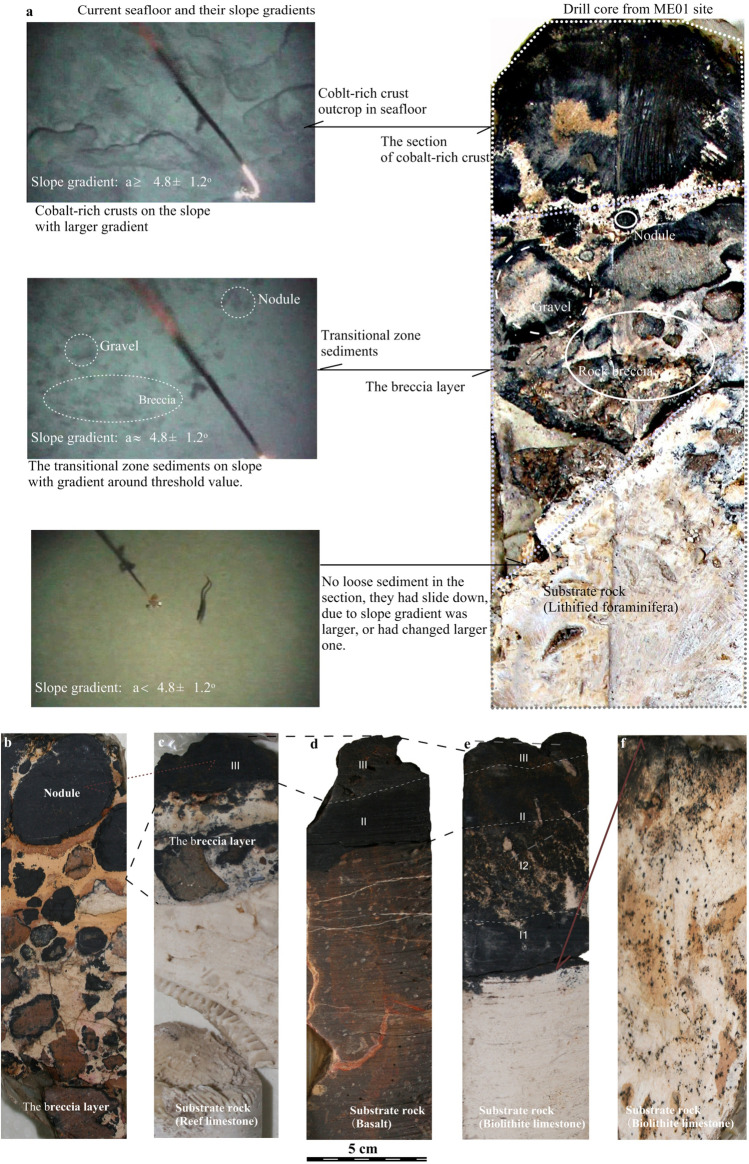
(**a**) The left panel shows seafloor images from the TV video survey line shown in Fig. 2a. The right panel is a sample section from a drill core at site ME01 (Fig. 2a), with cobalt-rich crust, a breccia layer and substrate rock appearing from top to bottom. The cobalt-rich crust and breccia layer are approximately 25 and 50 mm thick, respectively. The top two layers in the section appear to be accumulated by transitional zone sediments and cobalt-rich crust, which are shown in left images. Other sections of shallow drill cores are shown in (**b**–**f**) and will be discussed in the following section.

A core was obtained from the shallow borehole site labelled ME01 in Fig. 2a. Its section image is shown on the right side of Fig. 4a. In this sample, there is an approximately 5 cm thick consolidated intercalation of breccia, debris, ferromanganese nodules and gravel between the upper section of cobalt-rich crust and the substrate rock (i.e. lithified foraminiferal limestone). Mel’nikov et al.^43^ referred to this intercalation as buried ferromanganese nodules. In our work, we refer to this intercalation as the breccia layer between cobalt-rich crust and substrate rock. Twenty-four of the 205 drilling samples from the Magellan Seamounts, including six from seamount ME shown in Fig. 1b, have a breccia layer similar to ME01. Most importantly, those breccia layers have the same composition as transitional zone sediments and are 3–30 cm thick. Thus, we interpret the breccia layer as being buried transitional zone sediments. The stratigraphic relationship of cobalt-rich crust, which prefers larger gradient slopes, accumulated on transitional zone sediments, which prefer transitional gradient slopes (Fig. 3), observed in one drill core, suggests that the seamount slope topography has experienced adjustment over its geological history. The lack of loose sediments in the drill core suggests that if they were initially present, they have been lost due to mobilisation during the slope adjustment that resulted in the stratigraphic formation of cobalt-rich crust and transitional zone sediments.

### Stratigraphic chronology and records of slope changes in cobalt-rich crusts

There are layered structures in the cobalt-rich crust sections, e.g. layers III, II, I2 and I1 in Fig. 4c–e. Each layer has distinct textural features and stratigraphic chronology^42^. Several previous studies of stratigraphic chronology, such as isotope stratigraphy^44,45^, cobalt concentration^46^, a composite geochemical approach^47^ and cobalt-rich crust stratigraphy^48–50^, could be used to date cobalt-rich crusts on seamount slopes. Cobalt concentrations and the stratigraphy of cobalt-rich crusts are used together in the following discussion (Table 2). The average growth rate of the cobalt-rich crusts on the Magellan Seamounts slopes, which could be used to estimate the geological age of cobalt-rich crusts with known thicknesses, is approximately 4.7 mm/Myr. For instance, the cobalt-rich crust in sample ME01 is about 25 mm thick and belongs to layer III and thus is estimated that it began growing up to 5.3 Myr during the Pliocene.

**Table 2:** Stratigraphic chronology of cobalt-rich crusts on the Magellan Seamounts.

Dating by growth rate and thickness of cobalt-rich crust**				Dating by cobalt-rich layers***		
Seamount	Sample numbers*	Co (%)	Growth rate (mm/Myr)	Crust layer	Age (Myr)	Stratigraphic chronology
MA	32	0.51	4.7	III	1.0–5.0	Pliocene–quaternary
MC	18	0.52	4.6	II	6.0–23.0	Miocene
ME	55	0.50	4.9	I2	24.0–48.0	Oligocene, upper–middle Eocene
MK	78	0.51	4.7	I1	48.0–53.0	Low Eocene–upper palaeocene
Total	183	0.51	4.7	R	65.0	Upper Maestrichtian

*Samples obtained through drilling shallow boreholes and dredging, whole rock analysis by absorption spectrometry.

**Estimated by the formula of Puteanus and Halbach^49^.

***According to Melnikov and Pletnev^50^.

**Figure 5:**
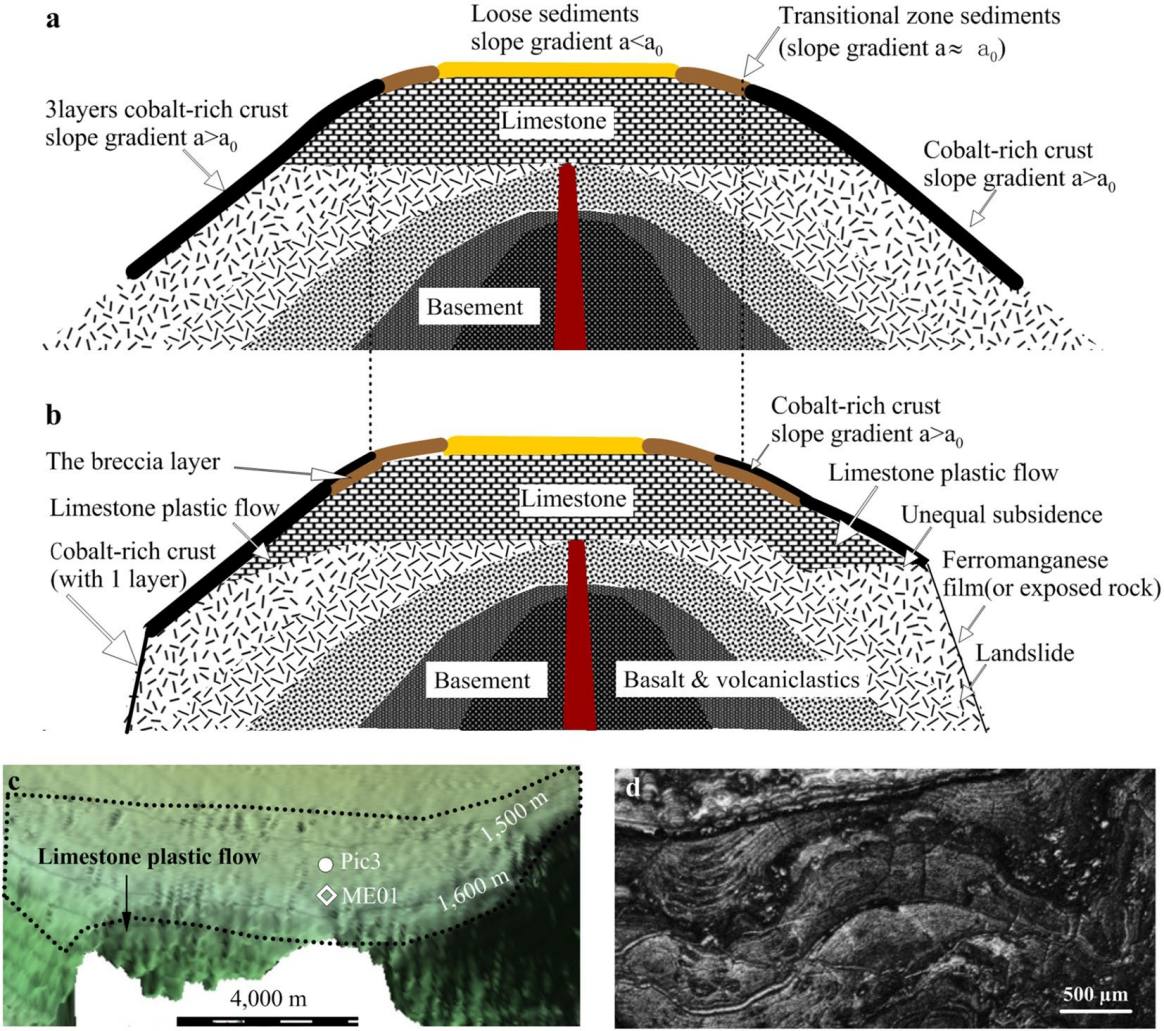
Extinct seamount evolution. (**a**) Seamount structure and depositions on its slopes. (**b**) Slope topography changed, and the depositional patterns adjusted accordingly. Gentle slope topography changes could be caused by unequal subsidence and plastic flow of rocks overlying the basement moving down slope. (**c**) Plastic flow of limestone on the slope shown in Fig. 2a. (**d**) Micro-deformation structures in the section of cobalt-rich crust overlying limestone that could be caused by plastic flow.

The slopes where the drill cores shown in Fig. 4b,c were obtained underwent a gentle slope adjustment, going from a gentle slope to larger gradient slope. By contrast, the slopes where the drill cores shown in Fig. 5d,e were obtained underwent sudden slope collapse, becoming steep and providing clear earlier deposits for the growth of subsequent cobalt-rich crusts. The slope where the drill core shown in Fig. 4b is located maintained a transitional zone slope topography for long time (approximately 5 Myr) and underwent deposition of transitional zone sediments that have since been consolidated. The slope where the drill cores shown in Fig. 4c are located is similar to that of ME01 and underwent a sequential slope gradient adjustment allowing deposition of transitional zone sediments and cobalt-rich crust. The slope where ME10 is located (Fig. 4e) underwent sudden slope collapse, forming a steep slope for long term (approximately 53 Myr), for Layers III, II, I2 and I1 to deposit on. The slope where ME43 sits (Fig. 4f) was deformed to an 18.9° gradient slope. This slope is too steep for loose sediments and transitional zone sediments to deposit on and was exposed to the seafloor too recently to allow ferromanganese film deposit.

### Seamount evolution and slope topography changes

The seamounts underwent several volcanic eruptions before reaching the extinct stage, causing different rock compositions to stack layer by layer, which generally formed the current seamount topography (Fig. 5). It suggested that the first stage layers consist mainly of basalts and that the second stage layers are made up of basalt and 20% volcaniclastics^51^. Volcaniclastics are the dominant rock types^52^ in the third stage, accounting for more than 60% and are deposited on flanks in the last stage^10^. When seamounts entered the extinct stage, subsidence due to the load of the seamounts themselves became the dominant vertical force^53^. For instance, the island of Hawaii has a subsidence rate of 2.6 mm/year^54,55^. When the seamounts subsided below sea level, biogenic limestone began being deposited during the Cretaceous (Aptian–Cenomanian), which continued for a long time. This limestone is 470–800 m thick and is mainly distributed between 800 and 2500 m deep^56^ (Fig. 5). Over time, seamount surfaces became encrusted with chemical colloid depositions, mainly cobalt-rich crusts. Meanwhile, pelagic sediments (referred to as loose sediments) were deposited on the slopes and mainly on the flat tops of summits (Fig. 5a).

Intrusive activities on seamounts are demonstrably major factors in their evolution, leading to slope collapse on seamount flanks^10^ and topography adjustment in slope gradients caused by plastic deformation^57–59^ or seamount subsidence.

We suggest that limestone on seamount slopes, especially on the summit boundaries (e.g. sites where the six cores containing breccia layers were collected on seamount ME shown in Fig. 1b) underwent plastic flow as described in previous studies^57–59^. Limestone had been moving down the slope under the pull of gravity at a very slow speed, e.g. 0.1 mm/year (the speed will be discussed in next paragraph). When plastic flow occurred, the limestone and overlying deposits (i.e. transitional zone sediments and cobalt-rich crusts) moved down the slope together, and some micro-deformation structures would be formed in the overlying rocks^60^. The micro-deformation structures in a cobalt-rich crust shown in Fig. 5d could be evidence of plastic flow. When the plastic flow moved to a different part of the slope, the gradient was altered accordingly.

As suggested by Fig. 5c, before 5 Myr, the limestone rock with overlying transitional zone sediments underwent plastic flow downslope from site Pic3 to site ME01 over a long period (up to 5 Myr), and the slope changed from a transitional gradient (a ≈ 4.8° ± 1.2°) to a steeper gradient (a > 4.8 ± 1.2°) accordingly, with cobalt-rich crust deposits overlaying the transitional zone sediments. The distance between sites Pic3 and ME01 is approximately 500 m; therefore, if the plastic flow took 5 Myr, then the plastic flow velocity can be estimated to be 0.1 mm/year.

Another factor involved in slope gradient changes could be unequal subsidence between different rock composition layers (right panel of Fig. 5a).

When collapse occurs on the flanks, previous deposits would be either cleared or covered, and bare rocks would be exposed to the seafloor, allowing subsequent deposition to develop. On steep slopes, old landslides deposited thicker cobalt-rich crusts (left panel in Fig. 5b) and later landslides deposited thin cobalt-rich crusts or exposed bare rocks to the seafloor without overlying deposits (right panel in Fig. 5b).

#### Model for depositional patterns constrained by topography changes

**Figure 6:**
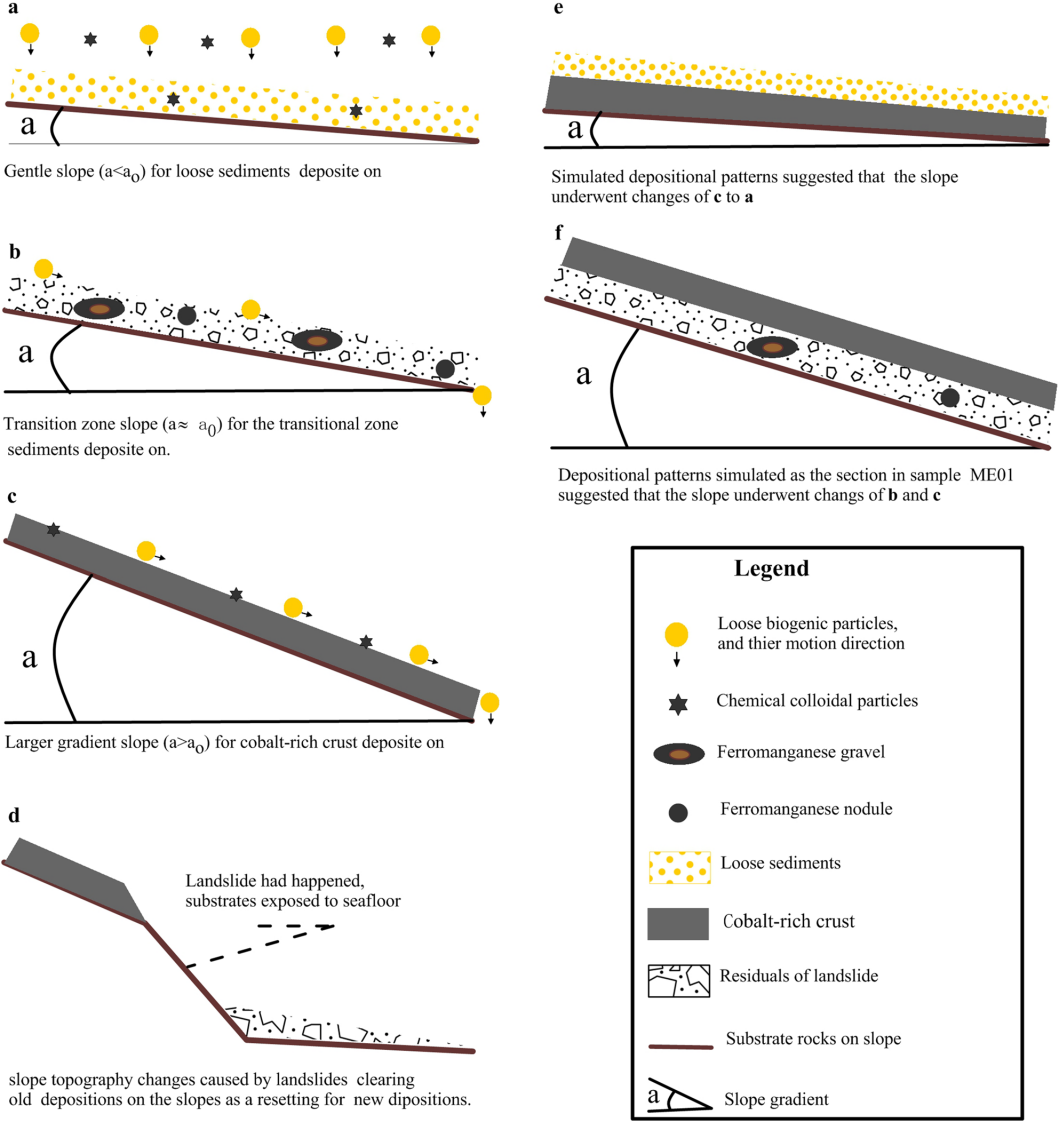
Deposition patterns constrained by changes to seamount slope topography.

1. Seamounts need to have gentle slopes to promote loose sediment deposition (referred to as *gentle slope for loose sediments*). Two types of deposit (i.e. loose biogenic particles and ferromanganese chemical colloids) are well preserved on gentle slopes or on the flat tops of seamounts with gradients less than the threshold value (a < a_0_). Loose biogenic particles are generally larger and are more matter flux, whereas ferromanganese chemical colloids are smaller and less matter flux. Therefore, these two types of sediment deposited on the gentle slopes together, with the former dominating the latter, and the depositional pattern presents as loose sediments (Fig. 6a).
2. When slope gradients achieve a threshold value (a ≈ a_0_), transition zone slope development, in which transitional zone sediments are deposited (referred to as *transition zone slope for transitional zone sediments*) can occur. Loose particles would be subject to higher rates of mobilisation and could be lost. By contrast, breccias, ferromanganese nodules and gravels that exhibit less tendency for mobilisation would remain on the slope. If the slope maintained this topography for a long time, the breccias and nodules would consolidate through some type of cementation (e.g. carbonate cementation), at which point transitional zone sediments would be preserved in the stratigraphic column. However, if slopes increase over time, transitional zone sediments do not have enough time to consolidate and will be removed. This pattern is shown in Fig. 6b.
3. Slopes with larger gradients (a > a_0_) promote deposition of cobalt-rich crust (referred to as *larger gradient slope for cobalt-rich crust*). Larger gradient slopes encourage the loss of loose sediments as their sliding friction is not enough to resist the pull of gravity. This exposes stable substrates, promoting chemical colloid deposition, mainly cobalt-rich crusts. This pattern is shown in Fig. 6c.
4. Intense slope topography collapses such as those resulting from landslides, slope collapse and volcanic activity could reset seamount slopes (referred to as *slope resetting for new depositions)*. This includes the removal or burial of prior deposits followed by deposition of new sediments. This pattern is shown in Fig. 6d.
5. The above scenarios are various static slope deposition patterns that can occur in different orders and combinations on a single slope over its geological history. Thus, the depositional patterns constrained by changes in topography would be explained by a combination of these scenarios. This model could be used to explain the depositional sequences observed in sections of samples taken from seamount slopes.

#### Several applications of the models

1. Slope topography has changed over time at the location of ME01. The section from sample ME01 shown in Fig. 4a has three stratigraphic layers, namely, an overlying cobalt-rich crust, a breccia layer and an underlying substrate rock layer. The cobalt-rich crust in layer III is 25 mm thick, suggesting that its formation began in the Pliocene. We thus speculate that the following process occurred. Approximately 5.3 Ma, the slope where ME01 is in Fig. 2a was a gentle slope or flat top seamount covered with loose sediments (*gentle slope for loose sediments*). The slope then changed from a gentle slope to a transition zone slope, which was accompanied by the loss of most of the finer grained components, lithification of larger particles and breccia rocks through chemical colloid deposition and burial of transitional sediments as the breccia layer (*transition zone slope for transitional zone sediments*). Lastly, the slope gradient continued to increase, resulting in deposition of a cobalt-rich crust (*larger gradient slope for cobalt-rich crust*). These processes are shown in Fig. 6f.
2. *Explanation of some sections in core samples* Additional processes such as *larger gradient slope for cobalt-rich crust* => *gentle slope for loose sediments* shown in Fig. 6e could explain hidden cobalt-rich crusts under loose sediment, and *larger gradient slope for cobalt-rich crust* => *gentle slope for loose sediments* => *larger gradient slope for cobalt-rich crust* might provide an alternative explanation for the interrupted growth of crusts found by Klemm et al.^61^ and Meng et al.^62^.

When slope topography changes occurred, the uppermost deposits might also change and be preserved within seamount slope stratigraphic records. Therefore, stratigraphic information obtained through drill cores might provide evidence of the geological history of slope changes. The models shown in Fig. 6 could thus be used to interpret sections of drill cores from slopes on all intraplate seamounts, including guyots and spire seamounts.

## Methods

### Positioning methods

A combination of on-board GPS and underwater acoustic positioning techniques allowed for a spatial resolution of relative position of up to 5 m.

### Bathymetry surveying and data processing

Bathymetric data were collected using an EM122 (Kongsberg, Inc.), a multibeam survey system that generates data that enables the production of wide-swath contour maps of the seafloor. Bathymetric data for all survey lines were processed manually on-board using Seafloor Information System software version 3.6. Post-processing consisted of editing the cross-track and navigation data (including the deletion of bad data, correction of position, etc.), leading to the creation of grid data. A 100 × 100 m grid size was selected because the raw data’s horizontal resolution was approximately 45–75 m, depending on the interval water depth of the studied region (1500 to 2500 m). The accuracy of the raw depth data is 3.0–5.0 m based on the following equation: depth accuracy = depth × 0.2%.

### Estimating slope gradients

A total of nine adjacent 100 m × 100 m grids are used to estimate the slope gradient of the sampling stations, which fall into the central grid using a 3 × 3 difference operator. This slope gradient value is simply the estimated value of the background slope in a 200 m × 200 m range of the sampling station.

### Slope gradient estimated error from surveying data

Assuming a slope gradient value of 4.8° and a depth error of approximately ± 4.2 m for depths of two locations separated by a horizontal distance of 200 m, the surveying data would yield an estimated error range of approximately 4.8° ± 1.2°, which is consistent with Fig. 3b.

### Seafloor TV video

The seafloor TV video technology constructed by COMRA was used to obtain images of the seafloor along a 15 km survey line along the southeast corner of seamount ME. The video camera was situated around 3–5 m from the seafloor and the video focus window on the seafloor is approximately 3 m × 5 m.

### Cobalt growth rate

Cobalt concentration analysis was conducted using absorption spectrometry. Thus, the age and growth rate of each cobalt-rich crust layer can be recalculated using the following formula: growth rate (mm/Myr) = 1.28/[Co (%) − 0.24]^49^. Related data are shown in Table 2.

### Drilling shallow borehole samples

The shallow boreholes were drilled using submersible rigs constructed by COMRA. Samples were obtained from 205 stations the distribution of sampling sites is shown in Fig. 1. Core sizes are 300–1000 mm long and 50 mm in diameter. Core samples were classified, and the thickness of their crusts was measured in the laboratory. Statistical parameters of the data are shown in Fig. 3.

### Section images of cores

Sample cores were divided into two parts with a chainsaw and then polished and cleaned with fine sandpaper. Section images were captured using a digital camera. The image of microdeformation structure in cobalt-rich crust is taken with a Zeiss microscope (Axioskop 40).

## Data Availability

The authors declare that the main data supporting the findings of this study are contained within the paper and are available in the Mendeley data (http://doi.org/10.17632/g83593d5jc.3). All other relevant data are available from authors upon reasonable request.
